# Akzeptanz psilocybin-assistierter Therapie in den deutschsprachigen Ländern

**DOI:** 10.1007/s00115-024-01792-5

**Published:** 2025-02-12

**Authors:** Nina Hartter, Marvin Däumichen, Christopher Schmidt, Max Wolff, Gerhard Gründer, Henrik Jungaberle

**Affiliations:** 1 Institut für Psychologie, Humboldt-Universität Berlin, Berlin, Deutschland; 2 MIND Foundation, Boxhagenerstr. 78, 12045 Berlin, Deutschland; 3 Klinik für Psychiatrie und Psychotherapie, Charité Universitätsmedizin Berlin, Berlin, Deutschland; 4 Zentralinstitut für Seelische Gesundheit, Abteilung für molekulares Neuroimaging, Medizinische Fakultät Mannheim, Universität Heidelberg, Mannheim, Deutschland; 5 OVID Clinic Berlin GmbH, Berlin, Deutschland; 6 OVID Tagesklinik GmbH, Berlin, Deutschland; 7 Standort Mannheim – Heidelberg – Ulm, Deutsches Zentrum für Psychische Gesundheit (DZPG), Mannheim, Deutschland

**Keywords:** Psychedelische Therapie, Psilocybin, Psilocybin-assistierte Therapien, Augmentierte Psychotherapie, Akzeptanz, Psychedelic therapy, Psilocybin, Psilocybin-assisted therapy, Augmented psychotherapy, Acceptance

## Abstract

**Hintergrund:**

Klinische Studien mit Psilocybin in Kombination mit Psychotherapie zeigen vielversprechende Ergebnisse bei der Behandlung verschiedener psychischer Störungen. In der Ärzte- und Psychotherapeutenschaft sowie unter Patient:innen bestehen auch Unwissen, Ablehnung und Vorurteile gegenüber dieser neuen Therapieform. Ziel dieser Studie war es, einen möglichst repräsentativen Eindruck vom Informationsstand und der Einstellung zur Implementation psilocybin-assistierter Therapie (PAT) unter Expert:innen für psychische Gesundheit sowie Patient:innen und der Allgemeinbevölkerung zu erheben.

**Methodik:**

Mittels einer Onlineumfrage wurden Informationen zu Einstellungen und Kenntnisstand von 1456 Teilnehmenden, darunter Ärzt:innen, Psychotherapeut:innen und Patient:innen, erfasst sowie Effekte der experimentellen Gabe von Informationen zu Potenzialen und Risiken getestet. Ergebnisse wurden mittels Varianzanalysen und Regressionsmodellen ermittelt.

**Ergebnisse:**

Regressionsanalysen zeigten, dass ein höherer Kenntnisscore und selbsteingeschätzter Kenntnisstand, eigene Behandlungserfahrung, aber auch eigene Erfahrung mit Psychedelika positivere Einstellungen bezüglich der Einführung von PAT vorhersagten (*F*[8, 1447] = 154,646, *p* < 0,001, R^2^ = 0,39). Die Vermittlung von Informationen über die Potenziale von PAT führte dann zu einer höheren Akzeptanz, wenn diese mit Informationen über deren Risiken kombiniert wurden.

**Diskussion:**

Die Ergebnisse zeigen, dass Teilnehmende einer Einführung psilocybin-assistierter Therapie optimistisch gegenüberstehen. Der Zusammenhang zwischen Wissen und Akzeptanz wurde bestätigt. Dass die Vermittlung ausgeglichener Informationen zu höherer Akzeptanz führt, sollte bei der Aufklärung und Berichterstattung über PAT berücksichtigt werden.

## Abstract (en)

**Background:**

Clinical studies with psilocybin in combination with psychotherapy show promising results for the treatment of various mental disorders; however, there still exists a lack of knowledge, rejection and prejudice towards this new form of therapy among doctors, psychotherapists and patients. The aim of this study was to gain a representative impression, as far as possible, of the level of information and attitudes towards the implementation of psilocybin-assisted therapy (PAT) among mental health experts, patients and the general population.

**Methods:**

An online survey was used to collect information on the attitudes and knowledge of 1456 participants and to test the effect of a brief intervention. Results were determined using analyses of variance and regression models.

**Results:**

Regression analyses showed that a higher knowledge score and self-assessed level of knowledge, own treatment experience and also own experience with psychedelics predicted more positive attitudes towards the introduction of PAT, *F*(8, 1447) = 154.646, *p* < 0.001, R^2^ = 0.39. Providing information about the potential of PAT only led to higher acceptance when combined with information about its risks.

**Conclusion:**

The results indicate that participants are optimistic about the implementation of PAT. The relationship between knowledge and acceptance was confirmed. The fact that receiving balanced information leads to higher acceptance should be taken into account when educating and reporting on PAT.

## Hintergrund

Psilocybin-assistierte Therapien (PAT) könnten zukünftig eine neue Behandlungsoption für psychische Störungen darstellen [30]. Für eine sichere und effektive Einführung von PAT in das deutsche Gesundheitssystem müssen die Einstellungen betroffener Personengruppen berücksichtigt werden. Mit dieser Studie erheben wir Daten zum Informationsstand und den Einstellungen zur Einführung von PAT unter Expert:innen für psychische Gesundheit, Patient:innen sowie der Allgemeinbevölkerung.

Arzneimittel aus der Substanzklasse *Psychedelika* wie Psilocybin nähern sich mit in den nächsten Jahren erwartetem Abschluss von Phase-III-Studien einer Zulassung im Arzneimittelmarkt [13]. Klinische Studien mit Psilocybin in Kombination mit Psychotherapie zeigen vielversprechende Ergebnisse für die Behandlung von Depressionen, Angststörungen und Substanzgebrauchsstörungen [1, 6, 10–12, 16]. Aufgrund der auffälligen subjektiven Wirkung von Psilocybin ist eine Verblindung in diesen klinischen Studien allerdings nicht vollständig zu erreichen [25, 27], was eine eingeschränkte Beurteilbarkeit Placebo-kontrollierter Studien zufolge hat.

Psychedelika-spezifische Risiken im Rahmen klinischer Studien wurden bisher nicht ausreichend erfasst sowie unzureichend definiert [5]. Schwere Nebenwirkungen, die mit rekreationalem Gebrauch von Psychedelika in Verbindung gebracht werden, wie psychotische Episoden, Suizidalität sowie halluzinogenbedingte anhaltende Wahrnehmungsstörungen („hallucinogen persisting perception disorder“, HPPD) können auch im Rahmen psilocybin-assistierter Therapien auftreten [20]. Allerdings sind unerwünschte klinische Ereignisse sowie langanhaltende Nebenwirkungen von Psilocybin vermutlich selten, wenn ausreichende Vorsichtsmaßnahmen umgesetzt werden [17, 28]. Psilocybin gilt als nicht abhängigkeitserzeugend und sicher und weist im Kontext einer ärztlich-psychotherapeutischen Begleitung in bisherigen Studien ein relativ gutes Risikoprofil auf [4, 18, 29].

Trotz wachsendem Interesse an psychedelischen Therapien bestehen in der Ärzte- und Psychotherapeutenschaft sowie unter Patient:innen mit psychischen Beschwerden auch Unwissen, Ablehnung und Vorurteile gegenüber dieser neuen Therapieform [4, 8, 9, 14, 29]. Laut Umfragestudien wird Psilocybin (u. a. aufgrund des rechtlich illegalen Status) als gefährlich und fälschlicherweise abhängig machend wahrgenommen [4, 8, 9, 14]. Diese negativen Bewertungen gehen möglicherweise auf einen geringen Kenntnisstand bezüglich psychedelischer Therapien unter Ärzt:innen und Psychotherapeut:innen zurück [9, 26]. Ein höherer Kenntnisstand zu PAT scheint mit optimistischeren Sichtweisen diesbezüglich zusammenzuhängen [2, 24, 29, 32]. In einer explorativen Vorstudie wurde ersichtlich, dass bereits kurze wissenschaftliche Informationen einen positiven Effekt auf die Einstellung von Expert:innen für psychische Gesundheit gegenüber Psilocybin erwirkten [29]. Diese Offenheit spiegelt sich auch in der Befürwortung weiterer Forschung und dem Wunsch nach Wissensförderung und klinischen Trainings zu psychedelischen Therapien wider [4, 14, 22, 26].

Das Ziel dieser Studie war es, einen möglichst repräsentativen Eindruck des Informationsstands und der Einstellung zur Implementation von Psilocybin in der Behandlung psychischer Störungen unter Ärzt:innen, Psychotherapeut:innen, Patient:innen sowie der Allgemeinbevölkerung zu erlangen. Außerdem sollten mögliche Zusammenhänge zwischen Einstellungen und demografischen Merkmalen der Teilnehmenden erfasst und der Effekt einer edukativen Kurzintervention getestet werden.

Wir vermuteten einen positiven Zusammenhang zwischen dem derzeitigen Kenntnisstand über psilocybin-assistierte Therapien und deren Akzeptanz. Wir gingen davon aus, dass solche Personen optimistischere Einstellungen zeigen, die einen hohen Kenntnisstand zu psilocybin-assistierten Therapien zeigen, sowie solche mit Eigenerfahrung psychedelischer Substanzen. Außerdem postulierten wir, dass das Lesen ausgeglichener Informationen über psilocybin-assistierte Therapien zu höherer Akzeptanz führt als das Lesen über jeweils nur Risiken oder nur Potenziale. Wir gingen davon aus, dass ärztliche und psychotherapeutische Berufserfahrung sowie Behandlungserfahrungen mit Psychosen mit geringerer Akzeptanz assoziiert sind, da Ärzt:innen und Psychotherapeut:innen mit zunehmender Erfahrung vermutlich häufiger mit Menschen in Kontakt gekommen sind, die durch rekreationalen Psychedelikagebrauch psychische Schäden erlitten haben, und dies möglicherweise mit einer erhöhten Skepsis gegenüber der therapeutischen Anwendung von Psychedelika einhergeht.

Weiterhin beinhalteten unsere Hypothesen, dass Personen, die in psychotherapeutischer Behandlung waren oder sind sowie Patient:innen, die mit ihrer bisheriger Behandlung unzufrieden waren, eine höhere Akzeptanz aufweisen.

## Methode

### Studiendesign

Die Onlineumfrage wurde mit dem Befragungstool SoSci Survey [21] erstellt. Je nach zufällig zugewiesener Experimentalgruppe erhielten die Teilnehmenden zu Beginn der Umfrage Informationen zu den Risiken von Psilocybin (1), zu den Potenzialen von Psilocybin (2), zu Risiken und Potenzialen (3) oder keine weiteren Informationen (4). Anschließend wurden neben demografischen Informationen die Selbsteinschätzung des Kenntnisstandes sowie der derzeitige Kenntnisstand anhand von sechs zu bewertenden Aussagen über Psilocybin ermittelt (Tab. 1). Zur Erfassung der Einstellungen wurden sieben Items im Likert-Format aus der Umfrage „A Survey of American Psychiatrists’ Attitudes Towards Classis Hallucinogens“ [4] ins Deutsche übersetzt, wobei der Begriff „hallucinogens“ durch „Psilocybin“ ersetzt wurde. Zusätzlich wurde die Offenheit bezüglich neuer Therapieansätze (*Offenheit für neue Therapieansätze*) sowie bezüglich PAT im Speziellen (*Offenheit für PAT*) erhoben (Tab. 2). Für eine Selbsteinschätzung der Akzeptanz wurden Teilnehmende gebeten, ihre Meinung zum therapeutischen Gebrauch von Psilocybin auf einer gleitenden Skala von 1 (starke Ablehnung) bis 100 (starke Befürwortung) auszudrücken. Schlussendlich wurde gefragt, ob und wie häufig die Teilnehmenden selbst verschiedene psychoaktive Substanzen konsumiert haben.

**Tab. 1:** Antworten auf die Kenntnisfragen bei 1456 Teilnehmenden

Kenntnisfrage (R) = richtig, (F) = falsch	Stimmt nicht *n* (%)	Stimmt *n* (%)	Weiß ich nicht *n* (%)
1. Im klinischen Umfeld ist die Sicherheit von Psilocybin gut zu gewährleisten (R)	76 (5,1)	1162 (77,8)	256 (17,1)
2. In der wissenschaftlichen Literatur gibt es Berichte von HPPD (fortbestehende Wahrnehmungsstörung nach Halluzinogengebrauch) im Kontext einer therapeutischen Verwendung von Psilocybin unter ärztlicher Aufsicht (F)	214 (14,3)	369 (24,7)	911 (61,0)
3. Die Einnahme von Psilocybin kann in therapeutischem Kontext Patient:innen den Zugang zu und die Akzeptanz von schwierigen Emotionen und Erinnerungen ermöglichen (R)	27 (1,8)	1335 (89,4)	132 (8,8)
4. Psilocybin kann leicht abhängig machen (F)	1207 (80,8)	103 (6,9)	184 (12,3)
5. Durch die Einnahme von Psilocybin kann es zur Auslösung einer psychotischen Episode kommen (R)	79 (5,3)	1072 (71,8)	343 (23,0)

**Tab. 2:** Zustimmung zu den Aussagen zur Akzeptanz (1–7), selbsteingeschätztem Kenntnisstand (8), Offenheitsitems (9–10) bei 1456 Teilnehmenden

Aussage	Stimme überhaupt nicht zu *n* (%)	Stimme eher nicht zu *n* (%)	Weder noch *n* (%)	Stimme eher zu *n *(%)	Stimme stark zu *n *(%)
1. Psilocybin erhöht das Risiko für spätere psychische Störungen	307 (20,5)	701 (46,9)	284 (19,0)	181 (12,1)	21 (1,4)
2. Die Anwendung von Psilocybin in der Behandlung psychischer Störungen ist vielversprechend	25 (1,7)	68 (4,6)	103 (6,9)	553 (37,0)	745 (49,9)
3. Psilocybin erhöht das Risiko für langfristige kognitive Beeinträchtigungen	568 (38,0)	593 (39,7)	224 (15,0)	98 (6,6)	11 (0,7)
4. Die Anwendung von Psilocybin im Rahmen einer Psychotherapie kann den Therapieerfolg steigern	28 (1,9)	54 (3,6)	112 (7,5)	551 (36,9)	749 (50,1)
5. Die Verwendung von Psilocybin für den nichttherapeutischen Gebrauch sollte verboten sein	517 (34,6)	412 (27,6)	228 (15,3)	184 (12,3)	153 (10,2)
6. Die Anwendung von Psilocybin in der Behandlung psychischer Störungen sollte weiter erforscht werden	14 (0,9)	19 (1,3)	28 (1,9)	163 (10,9)	1270 (85,0)
7. Die Anwendung von Psilocybin ist sogar unter ärztlicher Aufsicht unsicher	603 (40,4)	554 (37,1)	192 (12,9)	122 (8,2)	23 (1,5)
8. Ich bin über die Wirkungen und Nebenwirkungen von Psilocybin gut informiert	71 (4,9)	158 (10,9)	168 (11,5)	603 (41,4)	456 (31,3)
9. Wenn Sie wegen einer psychischen Störung in Behandlung wären/sind: Ich würde neue Therapieansätze ausprobieren, wenn es Hinweise auf deren Wirksamkeit und Sicherheit gibt	20 (1,4)	36 (2,5)	42 (2,9)	473 (32,6)	881 (60,7)
10. Wenn Sie wegen einer psychischen Störung in Behandlung wären/sind: Ich würde Psilocybin im Rahmen einer therapeutischen Behandlung unter ärztlicher Verordnung und Aufsicht einnehmen	92 (6,3)	71 (4,9)	43 (3,0)	284 (19,6)	962 (66,3)

### Stichprobe

Die Rekrutierung erfolgte über 12 von 182 kontaktierten Institutionen wie einigen Psychotherapeuten- und Ärztekammern verschiedener Bundesländer sowie über Fach- und Berufsverbände für Psychotherapie im deutschsprachigen Raum. Außerdem wurde die Umfrage über die sozialen Netzwerke „LinkedIn“, „Facebook“, „X“ und „Instagram“ verbreitet. Von 1828 teilnehmenden Personen beendeten 1494 (81,73 %) die Umfrage. Datensätze von 38 Personen mussten aufgrund eines Wohnortes außerhalb des deutschsprachigen Raumes ausgeschlossen werden. Dies resultierte in einer Stichprobe von *n* = 1456. Die Teilnehmenden waren im Durchschnitt 42,3 Jahre alt (*SD* = 13,56). 805 (55,3 %) der Befragten waren weiblich, 20 (1,4 %) nichtbinär (Tab. 3).

**Tab. 3:** Demografische Merkmale der 1456 Teilnehmenden

Merkmal	*n *(%)	Akzeptanzscore M (SD)	Kenntnisscore M (SD)
**Geschlecht**	–	4,10 (0,03)	2,56 (0,06)
Weiblich	805 (55,3)	4,16 (0,03)	2,62 (0,06)
Männlich	618 (42,4)	4,34 (0,16)	3,55 (0,35)
Nichtbinär	20 (1,4)	4,06 (0,20)	3,00 (0,44)
Keine Angabe	13 (0,9)	4,10 (0,03)	2,56 (0,06)
**Wohnort**			
Deutschland	1359 (93,3)	4,12 (0,72)	2,60 (1,59)
Österreich	50 (3,4)	4,30 (0,62)	2,82 (1,29)
Schweiz	47 (3,2)	4,05 (0,67)	2,55 (1,60)
***Subgruppe Gesundheitsexpert:innen***			
**Ärztlicher Bereich**	138 (9,5)	4,06 (0,73)	2,33 (1,76)
Medizinstudium mit Facharzt für Psychiatrie und Psychotherapie	63 (4,3)	4,03 (0,74)	2,29 (1,74)
Medizinstudium mit Facharzt für Psychosomatik und Psychotherapie	13 (0,9)	4,32 (0,61)	2,23 (1,88)
Medizinstudium mit Facharzt für Allgemeinmedizin	13 (0,9)	4,36 (0,46)	3,23 (1,01)
Medizinstudium mit anderem Facharzt	35 (2,4)	4,12 (0,66)	2,34 (1,80)
Medizinstudium ohne Facharzt	28 (1,9)	3,90 (0,82)	2,32 (1,83)
**Psychotherapeutischer Bereich**	481 (33,0)	3,91 (0,78)	2,26 (1,64)
Verhaltenstherapeutische Verfahren	276 (19,0)	3,86 (0,77)	2,19 (1,70)
Tiefenpsychologische Verfahren	238 (15,9)	3,94 (0,82)	2,38 (1,55)
Psychoanalytische Verfahren	62 (4,3)	3,79 (0,83)	2,02 (1,51)
Systemische Verfahren	68 (4,7)	4,05 (0,77)	2,37 (1,57)
Komplementärtherapien	111 (7,4)	4,08 (0,89)	3,21 (1,28)
**Andere Gesundheitsberufe**	149 (10,2)	4,18 (0,71)	2,91 (1,54)
***Subgruppe (ehemalige) Patient:innen***	718 (49,31 %)	4,19 (0,64)	2,65 (1,51)

### Statistische Verfahren

Die statistische Analyse der Daten erfolgte mit IBM SPSS Statistics. Das Signifikanzniveau wurde bei zweiseitiger Testung und nach Bonferroni-Korrektur für multiples Testen auf *p* < 0,003 festgelegt. Aus den Antworten auf die Kenntnisfragen wurde der Kenntnisscore gebildet, indem die Anzahl der falschen Antworten von der Anzahl der richtigen Antworten subtrahiert wurde. Der Akzeptanzscore nach Barnett et al. [4] ergibt sich aus dem Mittelwert der Antworten auf die sieben Aussagen zu Psilocybin (Tab. 2). Mit einem Cronbachs α von 0,86 weist dieser eine hohe interne Konsistenz auf.

Es wurden einfaktorielle Varianzanalysen sowie Regressionsanalysen durchgeführt, um den Einfluss der Experimentalgruppe sowie weiterer Variablen auf den Akzeptanzscore, die selbsteingeschätzte Akzeptanz, die Offenheit für neue Therapieansätze inklusive für PAT sowie auf die Bewertung von Psilocybin in der Behandlung spezifischer Indikationen zu testen. Als Prädiktoren in jeweiligen linearen Regressionsmodellen wurden die experimentelle Bedingung, demografische Merkmale (Alter, Geschlecht, Wohnort, Ausbildungsniveau, Vorgeschichte psychischer Störungen), die Selbsteinschätzung der Teilnehmenden zu ihrem Kenntnisstand, die Anzahl der Erfahrungen mit psychoaktiven Substanzen sowie die Antworten auf die Kenntnisfragen untersucht.

## Ergebnisse

### Deskriptive Statistiken

Ein Großteil der Kenntnisfragen wurde richtig beantwortet. Der durchschnittliche Kenntnisscore betrug M = 2,60 (SD = 1,59, Min = −4,00, Max = 6,00) und der durchschnittliche Akzeptanzscore M = 4,13 (SD = 0,72, Min = 1,00, Max = 5,00). Die Akzeptanzscores und Kenntnisscores der verschiedenen Subgruppen sind in Tab. 3 aufgelistet.

Die Mehrheit der Befragten stimmte der Aussage „Ich bin über die Wirkungen und Nebenwirkungen von Psilocybin gut informiert“ eher (41,4 %) oder stark (31,3 %) zu. Ein Großteil der Befragten empfindet die Anwendung von Psilocybin in der Behandlung psychischer Störungen als vielversprechend (86,9 %), denkt, dass die Anwendung von Psilocybin im Rahmen einer Psychotherapie den Therapieerfolg steigern könne (78 %), und befürwortet die weitere Erforschung (95,9 %; Tab. 2).

### Weitere Ergebnisse

**Abb. 1:**
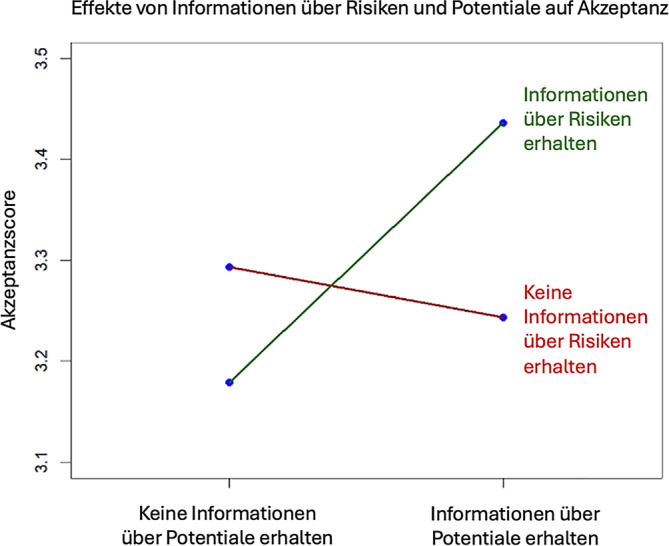
Interaktionsplot der Regression mit Informationen über Risiken und Potenziale sowie deren Interaktion als Prädiktoren

Durch eine ANOVA wurde kein Effekt der Experimentalgruppe auf den Akzeptanzscore sichtbar. Im Regressionsmodell des Akzeptanzscores (Tab. 4) zeigten sich Informationen über Risiken sowie die Interaktion zwischen Informationen über Risiken und Informationen über Potenziale als signifikante Prädiktoren. Wie in Abb. 1 zu erkennen, führen Informationen über Potenziale zu höherer Akzeptanz, wenn sie mit Informationen über Risiken kombiniert werden.

**Tab. 4:** Regressionsmodelle zu Einstellungen bei 1456 Teilnehmenden

	Akzeptanzscore nach Barnett et al. [4] (SE)	Selbsteingeschätzte Akzeptanz (SE)	Offenheit für neue Therapieansätze (SE)	Offenheit für PAT (SE)
***Gesamtstichprobe***				
Konstante	3,293*** (0,051)	43,118*** (2,413)	3,733*** (0,121)	2,765*** (0,166)
Informationen über Potentiale Koeffizient^a^	−0,050 (0,042)	N. s.	N. s.	N. s.
Informationen über Risiken Koeffizient	−0,114* (0,042)	N. s.	N. s.	N. s.
Informationen über Risiken * Informationen über Potentiale Koeffizient	0,143* (0,059)	N. s.	N. s.	N. s.
Selbsteingeschätzter Kenntnisstand Koeffizient	N. s.	6,441*** (0,657)	N. s.	0,276*** (0,035)
Kenntnisscore Koeffizient	0,269*** (0,009)	1,772*** (0,349)	N. s.	0,085*** (0,017)
Erfahrung Cannabis Koeffizient	N. s.	2,099*** (0,467)		0,118*** (0,026)
Erfahrung Psychedelika Koeffizient	0,057*** (0,009)	2,107*** (0,383)	0,060*** (0,016)	0,091*** (0,024)
Eigene Behandlung	0,106*** (0,030)	5,957*** (0,973)	0,178*** (0,039)	0,235*** (0,052)
F‑Wert	154,646***	60,451***	30,269***	49,481***
Korrigiertes *R*^*2*^	0,389	0,381	0,182	0,319
***(Ehemalige) Patient:innen***				
Konstante	3,909*** (0,148)	N. s.	4,474*** (0,047)	5,151*** (0,099)
Medikamentöse Behandlung Koeffizient	N. s.	N. s.	0,213*** (0,053)	N. s.
Zufriedenheit medikamentöse Behandlung Koeffizient	N. s.	N. s.	N. s.	−0,189*** (0,094)
F‑Wert	3,374*	N. s.	7,306***	12,517***
Korrigiertes *R*^*2*^	0,027	N. s.	0,026	0,074
	***Ärzt:innen***	***Psychotherapeut:innen***		
	**Selbsteingeschätzte Akzeptanz (SE)**	**Selbsteingeschätzte Akzeptanz (SE)**		
Konstante	94,960*** (4,366)	91,771*** (4,360)		
Berufserfahrung Koeffizient	N. s.	−7,989*** (1,338)		
Erfahrung mit Psychosen Koeffizient	−14,643*** (4,289)	N. s.		
F‑Wert	8,428***	9,290***		
Korrigiertes *R*^*2*^	0,140	0,080		

*p < 0,05, **p < 0,003, ***p < 0,001; *p*-Werte wurden nach Bonferroni-Korrektur angepasst (α = 0,003)

^a^Der Prädiktor Experimentalgruppe Potenziale wurde dem Regressionsmodell nach dem sukzessiven Ausschlussverfahren beigefügt. Die Koeffizienten sind unstandardisierte partielle OLS-Regressionssteigungen mit (robusten) Standardfehlern (*SE*) in Klammern

*N. s.* nicht signifikant

In den Regressionsmodellen sagte ein höherer Kenntnisscore eine höhere Akzeptanz, eine stärkere Befürwortung sowie eine höhere Offenheit für PAT voraus (Tab. 4). Ein höherer selbsteingeschätzter Kenntnisstand sowie eigene Erfahrung mit Cannabis sagten eine stärkere Befürwortung sowie eine höhere Offenheit für neue Therapieansätze voraus. Personen, die Erfahrung mit Psychedelika hatten, sowie Personen, die in Behandlung sind oder waren, wiesen eine höhere Akzeptanz, Befürwortung sowie eine höhere Offenheit für neue Therapieansätze und PAT auf. Personen, die selbst medikamentös behandelt wurden, zeigten sich eher bereit, neue Therapieansätze auszuprobieren, und Personen, die mit ihrer medikamentösen Behandlung unzufrieden waren, zeigten sich eher für eine Behandlung mit PAT bereit.

Ärzt:innen mit Erfahrung in der Behandlung von Psychosen zeigten eine geringere Befürwortung des therapeutischen Gebrauchs von Psilocybin. Je mehr Berufserfahrung Psychotherapeut:innen haben, desto geringer war die Befürwortung. Ärzt:innen hatten einen höheren Akzeptanzscore als Psychotherapeut:innen, F(3, 1452) = 29.845, *p* < 0,001, η^2^ = 0,058.

Der Großteil der Befragten befürwortete eine Behandlung mit Psilocybin bei den meisten der vorgestellten Indikationen: Depressionen (92,4 %), Angststörungen (74,6 %), posttraumatische Belastungsstörungen (69,4 %), Zwangsstörungen (62,9 %). Je höher der selbsteingeschätzte Kenntnisstand war, desto höher war die Befürwortung.

## Diskussion

Ziel der Umfrage war die Erfassung von Informationsstand und Einstellungen zur Implementation von Psilocybin in der Behandlung psychischer Störungen unter Ärzt:innen, Psychotherapeut:innen, Patient:innen sowie der Allgemeinbevölkerung. Zudem sollte der Zusammenhang zwischen Einstellungen und demografischen Merkmalen der Teilnehmenden untersucht sowie der Effekt einer Kurzintervention getestet werden.

Die Ergebnisse deuten darauf hin, dass die teilnehmenden Personengruppen einer Einführung psilocybin-assistierter Therapie optimistisch gegenüberstehen. Im Gegensatz zu früheren Umfragestudien [4, 29] weisen nur wenige Teilnehmende pessimistische Einstellungen bezüglich der Einführung von PAT auf. Dies spiegelt die Ergebnisse von Barnett et al. wider, die im Jahr 2018 eine Umfragestudie unter amerikanischen Psychiater:innen durchführten und in einer Folgestudie aus dem Jahr 2024 einen Trend zu positiveren Einstellungen feststellten [3]. In der ersten Studie stimmten der Aussage „The use of hallucinogens shows promise in treating psychiatric disorders“ 42,5 % der Teilnehmenden zu; im Jahr 2024 waren dies 80,9 % [3].

Die Teilnehmenden gaben außerdem an, selbst gut über PAT informiert zu sein. Auch das durch die Befragung gemessene Allgemeinwissen zu Psilocybin ist in unserer Stichprobe hoch. Wie erwartet sind Personen mit höherem Wissenstand optimistischer bezüglich der Einführung von PAT. Außerdem führt, wie erwartet, das Lesen ausgeglichener Informationen zu Risiken und Potenzialen von Psilocybin zu höherer Akzeptanz, d. h., wenn Informationen über Potenziale mit Informationen über Risiken kombiniert werden, führt dies zu höherer Akzeptanz als Informationen über Potenziale allein. Möglicherweise bieten sozialpsychologische Theorien aus der Überzeugungsforschung eine Erklärung für diesen Effekt. So besagt etwa die sog. „inoculation theory“ [7, 23], dass schwache (oder fehlende) Gegenargumente bei der empfangenden Person einen Widerstand erzeugen, der die Aufrechterhaltung bestehender Überzeugungen angesichts konträrer Informationen erleichtert. Die Gabe rein positiver Informationen könnte somit dazu führen, dass Teilnehmende, die im Voraus schon eine eher ablehnende Haltung gegenüber Psychedelika hatten, sich in dieser eher bestärkt fühlten. In zukünftigen Studien sollten auch die Effekte anderer Medien, wie z. B. Filme oder Aufklärungskampagnen, untersucht werden.

Analog zu den Ergebnissen früherer Studien [8, 26, 32] hängen eigene Erfahrungen mit Psychedelika der Befragten mit optimistischeren Sichtweisen bezüglich der Einführung von PAT zusammen. Es ist anzunehmen, dass durch das Erleben psychedelischer Erfahrungen deren therapeutischer Einsatz vorstellbar wird. In diesem Kontext ist jedoch auch das sog. „similarity bias“ zu nennen. Dieses beschreibt den Umstand, dass psychedelische Selbsterfahrung von Therapeut:innen zu einer geringeren Offenheit gegenüber andersartigen psychedelischen Erfahrungen ihrer Patient:innen und demnach zu möglicherweise restriktiven Therapieansätzen führen kann [15].

Es zeigt sich auch ein deutlicher Zusammenhang zwischen der Erfahrung eigener Behandlung aufgrund einer psychischen Störung und optimistischeren Sichtweisen. Eine mögliche Erklärung könnte sein, dass Menschen, die sich aufgrund eigener Behandlung mit ihrer psychischen Gesundheit auseinandergesetzt haben, möglicherweise mehr Verständnis für die Wirkungsweise neuartiger Therapieverfahren zeigen. Auch die Hoffnung auf Alternativen zu bestehenden Behandlungsmethoden könnte eine Rolle spielen. Hierfür spricht, dass Personen, die eine medikamentöse Behandlung erfahren haben und mit dieser unzufrieden waren, erhöhte Offenheit zeigen, sich Behandlungen mit neuen Therapieansätzen sowie im Speziellen Psilocybin zu unterziehen.

Ärzt:innen, die Erfahrung mit der Behandlung von Psychosen haben, zeigen eine geringere Befürwortung des therapeutischen Einsatzes von Psilocybin. Eine mögliche Erklärung könnte sein, dass die Effekte von Psilocybin in der medizinischen Lehre oftmals selektiv als psychoseinduzierend behandelt werden [19, 31] sowie dass nicht differenziert wird zwischen professionellen Psilocybinbehandlungen und einem nichtprofessionellem Eigenkonsum im rekreationalen Umfeld, der potenziell psychosefördernder sein kann.

Eine erhöhte psychotherapeutische Berufserfahrung hängt mit geringerer Befürwortung zusammen. Laut Barnett et al. [4] könnte dieser Befund auch so interpretiert werden, dass die Anzahl der Erfahrungen mit der Behandlung von Personen, die durch rekreationalen Substanzgebrauch psychische Schäden erlitten haben, mit zunehmender Berufserfahrung steigt und dies zu Ablehnung führt. Ärzt:innen zeigen eine höhere Akzeptanz als Psychotherapeut:innen, was in derer größerer Vertrautheit mit der Verabreichung von Medikamenten begründet sein mag.

Als Limitation der Studie ist die mangelhafte Repräsentativität der Stichprobe aufgrund der Rekrutierungsmethoden zu nennen. Die Akzeptanz unter den Teilnehmenden ist außergewöhnlich hoch, was an einem Selbstauswahlbias liegen könnte. Personen, die schon ein Interesse an PAT haben, neigen eher dazu, an einer Umfrage zu dieser Thematik teilzunehmen. Für die Rekrutierung einer repräsentativen Stichprobe mittels der Melderegister fehlten uns die zeitlichen Ressourcen. Für zukünftige Studien wäre ein solches Vorgehen zu empfehlen.

Zusammenfassend bestätigen die Ergebnisse der Umfrage den in vorherigen Studien festgestellten Zusammenhang zwischen Wissen und Akzeptanz psilocybin-assistierter Therapien. Das Vermitteln ausgeglichener Informationen scheint sich auf eine akzeptierendere Einstellung auszuwirken. Auf Fakten basierende Akzeptanz innerhalb der behandelnden Berufe und bei Patient:innen ist eine Voraussetzung für die erfolgreiche Einführung dieser Therapieform. Es ist zu erwarten, dass mit dem Fortschreiten und öffentlicher Berichterstattung über klinische Phase-II- und -III-Studien, wie der am Mannheimer Zentralinstitut für Seelische Gesundheit (ZI) und in der Charité Berlin stattgefundenen EPIsoDE-Studie [25], die Akzeptanz für diese Behandlungsverfahren den Ärzt:innen und Psychotherapeut:innen sowie unter Patient:innen und der Allgemeinbevölkerung weiter steigt.

## Fazit für die Praxis

- Befragte mit **höherem Kenntnisstand** über psilocybin-assistierte Therapien weisen eine **höhere Akzeptanz** für diese auf.
- Die Präsentation ausgeglichener Informationen zu Psilocybin führt zu höherer und das Präsentieren von Informationen zu Risiken zu geringerer Akzeptanz. Informationen über Potenziale führen zu höherer Akzeptanz, wenn diese mit Informationen über Risiken kombiniert werden.
- Befragte, die aufgrund einer psychischen Störung **in Behandlung** waren oder sind, weisen eine höhere Akzeptanz auf und Befragte, die mit ihrer **medikamentösen Behandlung unzufrieden** waren, weisen eine **höhere Offenheit** auf, psilocybin-assistierte Therapie selbst auszuprobieren.
- Ärzt:innen, die **Behandlungserfahrung mit Psychosen** haben, zeigen eine geringere Befürwortung für den therapeutischen Gebrauch Psilocybins.

## Data Availability

Die in dieser Studie analysierten Datensätze sind auf Anfrage bei der Autorin erhältlich.
